# Synthesis of Nanocrystalline SnO_x_ (x = 1–2) Thin Film Using a Chemical Bath Deposition Method with Improved Deposition Time, Temperature and pH

**DOI:** 10.3390/s111009207

**Published:** 2011-09-27

**Authors:** Saeideh Ebrahimiasl, Wan Md. Zin Wan Yunus, Anuar Kassim, Zulkarnain Zainal

**Affiliations:** 1 Institute of Advanced Technology, University Putra Malaysia, 43400, UPM Serdang, Malaysia; E-Mail: zulkar@science.upm.edu.my; 2 Department of Chemistry, University Putra Malaysia, 43400, UPM Serdang, Malaysia; E-Mail: anuar@science.upm.edu.my

**Keywords:** nanocrystalline, semiconductor, chemical bath deposition, photoluminescence

## Abstract

Nanocrystalline SnO_x_ (x = 1–2) thin films were prepared on glass substrates by a simple chemical bath deposition method. Triethanolamine was used as complexing agent to decrease time and temperature of deposition and shift the pH of the solution to the noncorrosive region. The films were characterized for composition, surface morphology, structure and optical properties. X-ray diffraction analysis confirms that SnO_x_ thin films consist of a polycrystalline structure with an average grain size of 36 nm. Atomic force microscopy studies show a uniform grain distribution without pinholes. The elemental composition was evaluated by energy dispersive X-ray spectroscopy. The average O/Sn atomic percentage ratio is 1.72. Band gap energy and optical transition were determined from optical absorbance data. The film was found to exhibit direct and indirect transitions in the visible spectrum with band gap values of about 3.9 and 3.7 eV, respectively. The optical transmittance in the visible region is 82%. The SnO_x_ nanocrystals exhibit an ultraviolet emission band centered at 392 nm in the vicinity of the band edge, which is attributed to the well-known exciton transition in SnO_x_. Photosensitivity was detected in the positive region under illumination with white light.

## 1. Introduction

Tin dioxide is an n-type wide-band gap semiconductor (E_g_ = 3.6 eV at 300 K) where inherent oxygen vacancies act as an n-type dopant [1,2]. Research on SnO_2_ attracts a lot of interest due to its many applications, such as in transparent electrodes, far-infrared detectors and high-efficiency solar cells [3,4]. It was recently reported that nanocrystalline SnO_2_ has different characteristics from bulk crystals. Nanocrystalline SnO_2_ thin films have also garnered attention since higher quality synthesis of SnO_2_ thin films was achieved.

A variety of methods, such as gas sensors [5], vacuum evaporation [6], chemical vapor deposition [7] and modified successive ionic layer adsorption and reaction [8] have been employed to prepare SnO_2_ thin films or nanoparticles. In the present investigation, nanocrystalline SnO_2_ thin films are prepared by using a simple chemical bath deposition (CBD) method. CBD, which is well known as a low temperature aqueous method for directly depositing large-area thin films of semiconductors has advantages over other techniques because it allows films to be deposited on substrates that might not be chemically or mechanically stable at high temperatures [9]. Moreover, CBD does not require sophisticated instruments such as vacuum systems. The starting chemicals are inexpensive and readily available and the parameters are easily controlled. Films deposited by this technique are now being developed for use in solar energy and other photonic applications, such as dye-synthesized solar cells [10], photothermal and photovoltaic conversions [11]. Decreasing the temperature and time for saving energy and a shift toward noncorrosive pH are the main objectives in chemical synthesis of TCO (transparent conductive oxide) for industry.

There are some literature reported the preparation of SnO_2_ films using chemical methods, and most of the films prepared by these methods need high temperatures or long deposition times and are prepared at corrosive pHs [12,13]. In this paper we prepared SnO_2_ films by a chemical bath deposition method in which a novel chelating reagent, triethanolamine, was used and deposition was conducted in a water bath to decrease the deposition time and temperature. Furthermore, the pH value was shifted to a noncorrosive region in this method. The optical, electrical and compositional characterization help to hasten the study of tin oxide potential application in transparent nanoelectrodes and transparent conductive oxide thin film solar cells.

## 2. Experimental Section

### 2.1. Preparation of SnO_x_ Thin Films

The substrates used for the deposition of SnO_x_ thin films were 76 × 25 mm^2^ sized glass slides. Prior to deposition, the glass slides were degreased with ethanol (0.1 M), etched with HCl solution (0.1 M) for 30 min and ultrasonically cleaned with deionized water for 50 min. Aqueous solution of 0.1 M tin chloride dihydrate, 0.5 M hydrogen peroxide, and complexing agents (0.15 M triethanolamine and 0.1 M ethylenediaminetetraacetic acid) were used to deposit SnO_x_ thin films. Twenty mL of tin chloride dihydrate solution was mixed with 3.0 mL triethanolamine in a 100 mL beaker. A homogenous solution was obtained after stirring for several minutes. With continuous stirring, 5 mL ethylenediaminetetraacetic acid and 5 mL H_2_O_2_ were added. Deionized water was added to make the volume up to 50 mL. The pH values of the samples were adjusted to 2.0, 5.0 and 7.0, using ammonia and HCl solutions. Prior to deposition, substrates were heated to 120 °C and quickly mounted in the cold reaction solution. The reaction vessels were placed in a water bath at 30 °C for 30 min. The samples were removed from the water bath, and dried at room temperature.

### 2.2. Characterization Techniques

The composition and structure of the films were characterized by X-ray diffraction analysis using a Philips PM 1730 diffractometer from 20° to 80° with CuKα1 radiation (λ = 0.15405 nm). The surface morphology of the deposited films was studied using atomic force microscope (Quetint-250). The chemical composition of SnO_x_ films was analyzed by an energy-dispersive X-ray analyzer (EDX) LEO 1455 VPSEM with Oxford Inca software. The optical transmission data in the wavelength range of 200–800 nm was recorded by a Lambda 2S Ultraviolet/Visible Spectrophotometer at room temperature. Thermogravimetric analysis of the powder of SnO_x_ nanocrystalline was obtained by a TGA Perkin Elmer Thermal Analyzer. The samples were scanned at room temperature to 600 °C at a heating rate of 10 °C/min in the presence of nitrogen (50 mL/min). The photoluminescence properties were studied at room temperature using a Perkin Elmer LS-55 analyzer. The current-voltage characteristics in the dark as well as illumination (tungsten-halogen lamp with intensity of 100 W/Cm^2^), were measured by an ADCM 6243 DC voltage current source/monitor.

## 3. Results and Discussion

### 3.1. Composition Analysis

**Figure 1.**
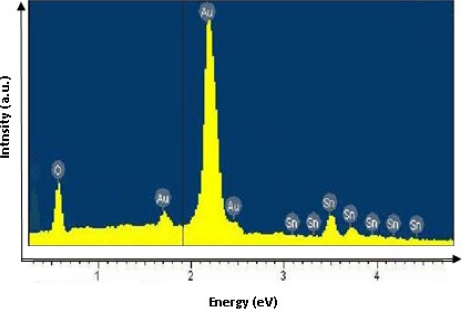
The EDX spectrum of nanocrystalline SnO_x_ thin film.

EDX was used to estimate the composition of the SnO_x_ thin films. Figure 1 shows the EDX spectrum of SnO_x_ thin film obtained by chemical bath deposition. The Au coating is reflected in the strong Au peak. The result illustrated in Figure 1 indicates the presence of oxygen and tin with a typical O/Sn ratio of 43/25 (or 1.72) which is close to the stoichiometry of the compound SnO_2_. SnO_2_ in its stoichiometric form acts as an insulator, but in its oxygen-deficient form tin dioxide behaves as an n-type semiconductor and the conductivity is thought to be due to intrinsic defect formation [14].

### 3.2. Structural Studies

**Figure 2.**
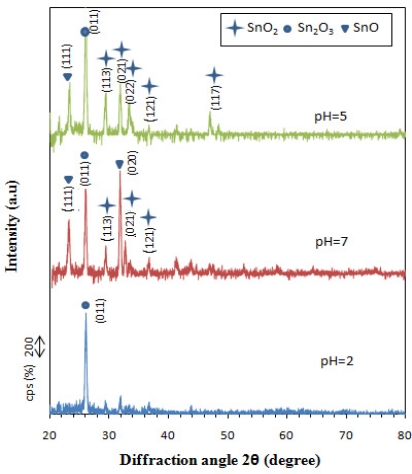
X-ray diffraction pattern of SnO_x_ thin film at various pH levels.

The XRD pattern of SnO_x_ thin films (Figure 2) deposited by chemical bath deposition method for 30 min indicate that they are of a polycrystalline nature. From the XRD patterns several SnO_2_ peaks are detected for the samples prepared at pH 5.0 and 7.0. The observed d-values are in good agreement with the Joint Committee on Powder Diffraction Standard (JCPDS) data for the orthorhombic structure of SnO_2_ (reference code: 078-1063). The Miller indices are shown above the diffractions. For the sample deposited at pH of 5.0 the strongest peak is at 2θ of 31.8° (corresponding to (021) reflection). It indicates that the preferred orientation lies along (021) plane. The other smaller peaks were at 2θs of (29, 33.3, 36.6, and 47) corresponding to the (113), (022), (121) and (117) planes, respectively. The crystallite size on the film was calculated by using Scherrer's formula for the (021) peak for 2θ of 31.8° and was found to be 36 nm.

For the film deposited at pH of 7.0 some SnO peaks were also detected with high intensity, indicating the presence of side product and due to the non-optimum conditions used for the deposition of the SnO_2_ thin film. For the film deposited at pH of 2.0 no intense SnO_2_ peak was detected.

### 3.3. Surface Morphology

**Figure 3.**
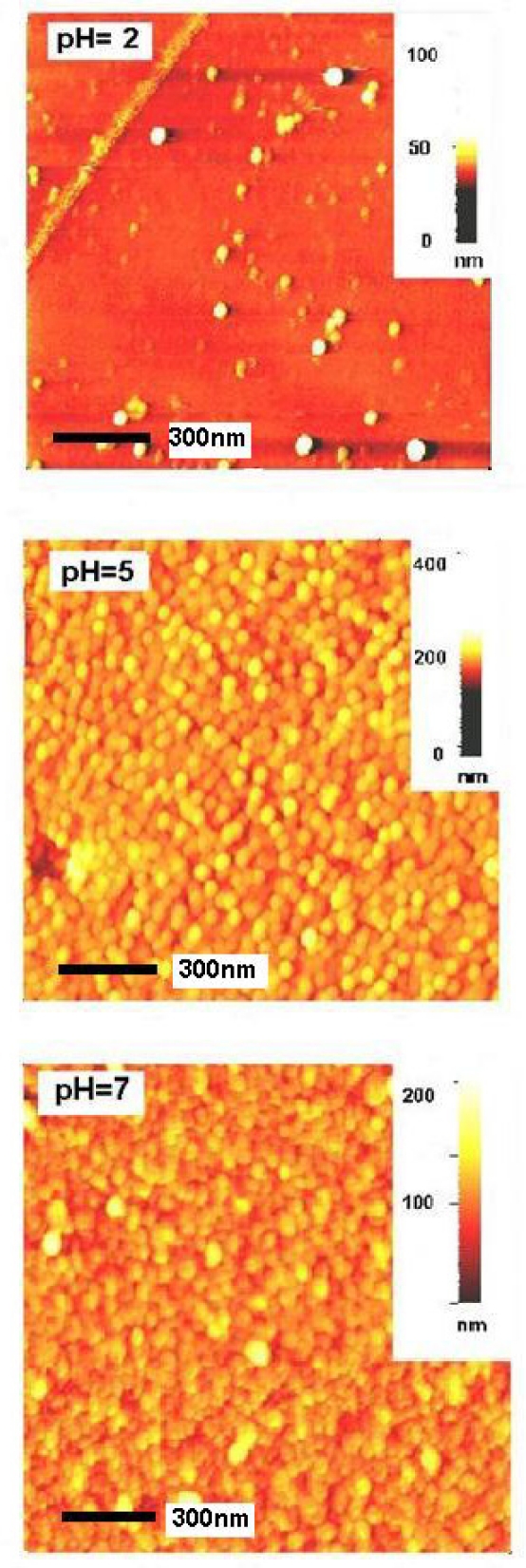
AFM images of nanocrystalline SnO_x_ thin film deposited at various pH levels.

Film morphology was examined using atomic force microscopy (AFM). Figure 3 illustrates the two-dimensional images of the films deposited at various pH levels. Figure 3 reveals incomplete growth of the films deposited at pH of 2.0 and uniform growth at pH of 5.0 or 7.0.

The formation of spherical, compact and nano sized grains on an amorphous background on films deposited at pH of 5.0 is an indication of nucleation by multiple growths. No pinholes or cracks were seen in the sample. The thickness of the film was 230 nm. The surface roughness of the film was about 8.74 nm, which is due to nucleation of grains by multiple growths that increases the incident light trapping effect of transparent tin oxide thin film for solar cell applications.

### 3.4. Optical Properties

**Figure 4.**
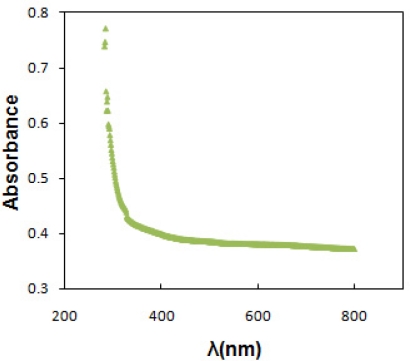
Absorption spectrum of nanocrystalline SnO_x_ thin film (pH = 5.0).

**Figure 5.**
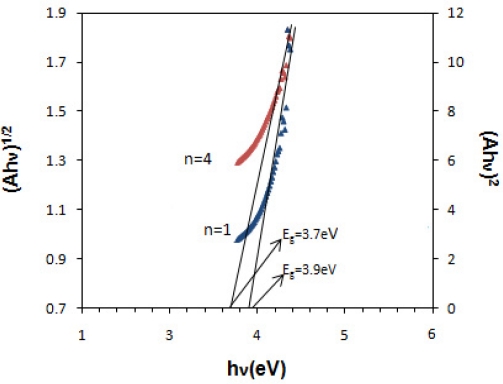
The plot of direct and indirect transition of the nanocrystalline SnO_x_ thin film (pH = 5.0).

Optical absorption was utilized to estimate band gap and type of optical transition. Figure 4 shows the absorption spectrum *versus* wavelength of SnO_x_ thin film deposited on glass substrate at pH of 5.0 and temperature of 30 °C. The optical data was then analyzed using the Stern equation for near-edge absorption:

$$\text{A}=\frac{\text{K}{\left(\text{h}\upsilon -{\text{E}}_{\text{g}}\right)}^{\text{n}/2}}{\text{h}\upsilon }$$

where K is constant, E_g_ is the energy separation between the valence and conduction band that called band gap, n is a pure number, that is equal to 1 for direct and 4 for indirect band gap semiconductors. Figure 5 shows the (Ahν)^2^ and (Ahν)^1/2^ plots of the SnO_x_ thin film for direct and indirect transition, respectively.

The band gap value were determined from the intercept of the straight line portion in maximum absorbance of the (Ahν)^2/n^ against the hν on the hν axis. In this case, the band gap of 3.9 eV and 3.7 eV was estimated for direct and indirect transition between valence and conduction bands of SnO_x_ thin film. The wide band gap and minimum absorbance of deposited SnO_x_ thin film in the UV-Visible range indicates the potential application of prepared thin films as window layer in solar cell device [3]. Table 1 shows the structural and optical characteristics of deposited SnO_x_ nanocrystalline thin film on glass substrate.

**Figure 6.**
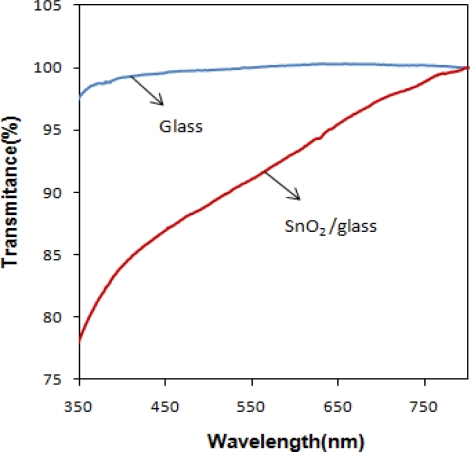
Optical transmittance spectrum of nanocrystalline SnO_x_/glass and glass slide (pH = 5.0).

High transmittance at visible-light wavelengths is a key factor for the semiconducting material in a transparent device, such as the SnO_2_ thin film solar cell or transistor. Figure 6 compares the transmittance of the SnO_x_/glass thin film and glass slides. The glass used has 96% optical transmittance in the visible part of the spectrum. The average transmittance for SnO_x_/glass in the visible part of the spectrum is comparable with glass and is more than 82%. This indicates that the deposited SnO_x_ thin film can be used as a transparent conductive oxide layer in solar cell devices.

### 3.5. Photoluminescence

**Figure 7.**
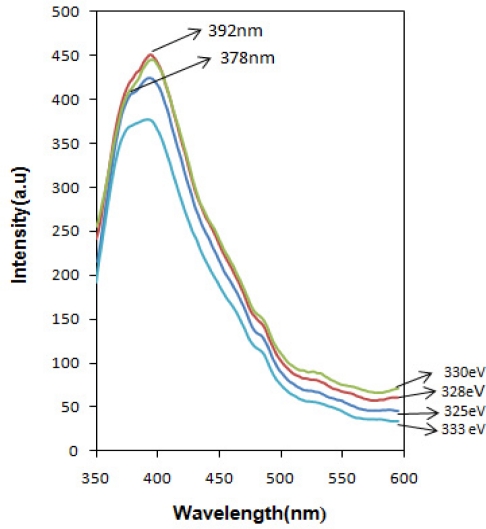
Photoluminescence spectrum of nanocrystalline SnO_x_ thin film excited with various photon energy (pH = 5.0).

Photoluminescence (PL) spectra of the SnO_x_ nanocrystalline were obtained in the wavelength range from 350 to 600 nm. The room temperature optical luminescence (Figure 7) from SnO_x_ nanocrystalline excited with various photon energies exhibit a strong emission band at 392 nm (3.17 eV) and a shoulder in 378 nm (3.28 eV). The expected near band edge was detected around 345 nm for direct transition and 335 nm for indirect transition, which is originated from exciton recombination of electron in the Sn ‘4d’ conduction band with a hole in the O ‘2p’ valence band. The observed shift to the visible range can be attributed to the recombination of photo-generated holes with singly ionized charge states in intrinsic defects such as oxygen vacancies, Sn interstitials, or impurities [15–17].

### 3.6. Photosensitivity

**Figure 8.**
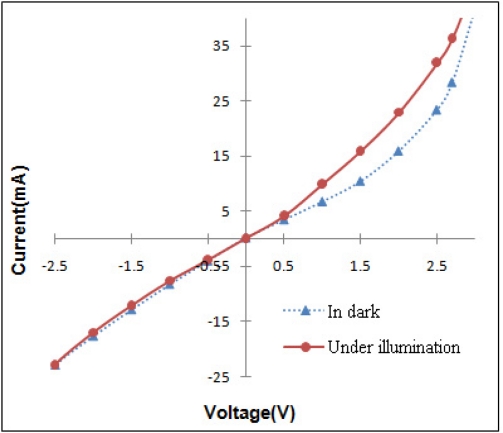
Photosensitivity of nanocrystalline SnO_x_ thin film in dark and under illumination (pH = 5).

As thin oxide films are expected to be semiconductors they should be sensitive to the light, with energy higher than their E_g_ and show a photocurrent in the region corresponding to its minority carrier current flow. Figure 8 shows the photosensitivity of the SnO_x_ thin film in the dark and under illumination. The lower value corresponds to the dark current, while the upper value of the current corresponds to the photocurrent when the sample, employed as anode, was illuminated. The fact that the photocurrent occurs on the positive potentials region indicates that electrons are minority carriers of the film and their concentration was then enhanced by illumination. Thus, the films prepared are n-type semiconductor and can be deployed as photoanode in the PECs (photoelectrochemical cells) application to facilitate oxidation on the electroactive species in the solution.

### 3.7. Thermogravimetric Analysis

**Figure 9.**
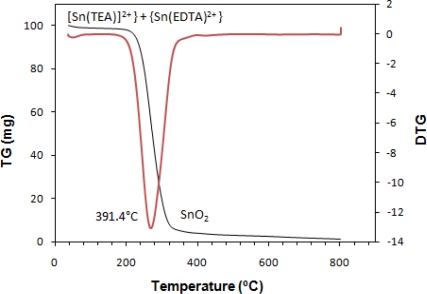
TG and DTG curve of SnO_x_ powder (pH = 5.0).

TG-DTG (thermogravimetry-derivative thermogravimetry) analysis was conducted to characterize the effects of heat treatment on the powder obtained from drying of precursor solution for nanocrystalline SnO_x_. However the characteristics of powder and film were not exactly the same, the heat treatment temperature can be obtained from the TG-DTG analysis. Figure 9 is the results of thermogravimetric measurements for prepared SnO_x_ nanocrystalline.

The weight loss for nanocrystalline SnO_x_ occured at a temperature between 184 °C to 269.2 °C, which is related to the decomposition of TEA from the [Sn(TEA)]^2+^complex and evaporation of solvent. It shows a residue retention of 3.9% at T > 320 °C. It can be concluded that to complete the crystallization of SnO_2_ and evaporation of complexing agent and water, the heat treatment should be conducted over 269.2 °C.

## 4. Conclusions

A simple chemical bath deposition technique was successfully utilized to synthesize nanocrystalline SnO_x_ thin films with decreased time of deposition and bath temperature and increased pH to the noncorrosive region by using TEA as a complexing agent. The preferred orientation lies along the (021) plane. AFM analysis showed a compact texture with small uniform grains of about 36 nm and 230 nm thick for the film deposited at pH of 5.0. EDX revealed that the average O/Sn atomic percentage is 1.72. Optical data analysis illustrated a direct band gap at 3.9 eV and an indirect transition at 3.7 eV. The wide band gap (3.9) and high transmittance (>82%) makes it possible for these thin films to be used in solar cell devices as transparent conductive oxide layers. The photoluminescence spectra of the nanocrystalline SnO_x_ exhibited a strong UV exciton peak at room temperature. The temperature for heat treatment of the as deposited thin film was found to be above 391 °C using TGA analysis.

## Figures and Tables

**Table 1.** Characteristics of prepared nanocrystalline SnO_x_ thin film in different pH.

**pH**	**Crystalline structure**	**t (nm)**	**R_a_ (nm)**	**E_g_(eV)**		**Size (nm)**	**T (%)**	**PL**	
				**Direct transition**	**Indirect transition**			**Intensity %**	**Position (nm)**
=5	orthorhombic	230	8.74	3.9	3.7	36	>85	445	392
=7	orthorhombic	180	17.1	4	3.8	52	>76	410	390
=2	orthorhombic	20	1.46	3.9	3.7	500	>98	320	392

Note: t = thickness, T = transmittance, R_a_ = surface roughness.
